# Supplementary material for: Spring Wheat’s Ability to Utilize Nitrogen More Effectively Is Influenced by Root Phene Variation
Source: Plants (Basel). 2023 Feb 23;12(5):1010. doi: 10.3390/plants12051010 (PMC10005382; doi:10.3390/plants12051010)
Supplement: Supplementary file 1 [file plants-12-01010-s001.zip › plants-1858239-supplementary.pdf]

Supplementary table:

Table S1: List of 175 genotypes under study

S.N.	Pedigree/ Genotypes	S.N.	Pedigree/ Genotypes
1	CL1449/HUW585	43	HD2948/HD2894
2	DL672/PS6270//DE8	44	PS755/HD29
3	HW5015/UP2538	45	PS755/PBW502
4	HD2898/HD29	46	CL1705/HD2894
5	HD2953/HS365	47	CL1705/HD2894
6	CL1449/PBW343	48	HD2851/HD2329
7	HD2878/HD29	49	HD2851/HD2329
8	CL2636	50	VL610/KUNDAN
9	PBW343/HD2877	51	PBW343/PICCI LOCAL//RL6080
10	18-HRWYT-214	52	HW3083/HD2639
11	18-SAWYT-303	53	HW3083/HD2639
12	HD2448/HW1305	54	HW3083/HD2639
13	HW4023/HW5028//HD2932/DW1309	55	HW3083/HD2639
14	HD2833/DW538	56	DWG107/RAJ 3765
15	39th IBWSN-23	57	DWG107/RAJ 3765
16	29th SAWSN-3145	58	WH542/UP290//DBW72
17	CSW 43	59	CL 2596/K9451//Cl882/HD2329
18	HD2932/UP2425	60	DWG107/HDK-10//C306
19	WL462/VCC/KOCL/3/Pes/Me-11	61	DWG107/HDK-10//C306
20	UP2425/CL146	62	DWG107/HDK-10//C306
21	JOSHI 3	63	HD3239/WR562
22	HD2967/DW1350	64	DL5/PBW343//DEEPALI
23	HD2448/HW1305	65	DL5/PBW343//DEEPALI
24	HD2878/HD29	66	DL5/PBW343//HD2891
25	CL1705/PBW12	67	DL5/PBW343//DEEPALI
26	CL1705/HD2894	68	DL5/PBW343//HD2891
27	HD2824/VL796	69	DL5/PBW343//HD2891
28	VL610/KUNDAN	70	DL5/PBW343//HD2891
29	31-ESWYT-132	71	DL5/PBW343//HD2891
30	5-EBWYT-519	72	CL2596/K9451//CL882/HD2009
31	43-IBWSN-1006	73	G-9/ MC-10
32	43-IBWSN-1077	74	G-9/MC-10
33	43-IBWSN-1090	75	CL22596/K9451//CL882/HD2329
34	43-IBWSN-1102	76	CP264/CL1633//CNO 63/WELLINGTON
35	43-IBWSN-1106	77	CL 2596/K9451//Cl882/HD2329
36	43-IBWSN-1153	78	CL2596/K9451/CL882//HD2009
37	43-IBWSN-1182	79	CL2596/K9451/CL882//HD2009
38	43-IBWSN-1187	80	HD2669/HD3016
39	29-SAWN-3028	81	HD2669/HD3016
40	HW5028/HD2250	82	HD2967/HUW631
41	HW2930/DW1309	83	HD2967/DBW17
42	HD2948/HD2894	84	HD2967/DBW17

85	HD2967/HD3024	128	HD 3090
86	HD2967/HD3024	129	HD 3059
87	HD2967/HD3024	130	HD 3086
88	HD2967/DBW54	131	GW 366
89	HD2967/DBW56	132	WL 711
90	HD2967/K-07-08	133	K 9107
91	HD2967/WH1073	134	43IBWSN-1201
92	HD2967/WH1073	135	28SAWSN-3012
93	HD2967/PBW 617	136	43-IBWSN-1054
94	HD2967/PBW 596	137	43-IBWSN-1187
95	HD2967/DT2756	138	28-SAWSN-3028
96	HD2967/HD3024	139	CL1705/HD2894
97	HD2967/HD3034	140	HW3083/HD2873
98	HD2967/HD3034	141	HD3117
99	HD2967/HD3035	142	CSW16
100	HD2967/HD3035	143	DL849/YSCN-08
101	HD2967/DT2761	144	DL672/P66.270//DE894/3/CUMYN
102	HD2967/DT2761	145	HD2329/WR544//PBW343/NW2041
103	HD2967/DT2761	146	18SAWYT-311
104	HI 1544	147	18HRWYT-214
105	HI1563	148	18th SAWYT 36
106	HI 1500	149	HD2733/PBW343/HD2733/PBW343
107	HI1531	150	WH542/YSCN-10
108	RAJ 4120	151	HD2687/HP1896//WH542
109	RAJ 3765	152	CSW44
110	RAJ 4083	153	PBW12/HW2078
111	DBW-14	154	HD2824/VL796
112	DBW17	155	HD2402/CPAN4067/HW4022/DW5247
113	WH 542	156	UP2425/UP2626
114	PBW 621-50	157	HD2733/HD2329
115	PBW 596	158	VL849/UP2571
116	PBW 590	159	HD2733/PBW343/HD2733/PBW343
117	PBW 502	160	IBWSN-06-07 KARNAL
118	PBW 443	161	HD2877/HS451
119	PBW 373	162	CL1591/CL1475
120	PBW 343	163	31 ESWYT121
121	HD 2285	164	6th EBYT-503
122	HD 2894	165	CL1705/HD2687
123	HD 2932	166	CL1705/HD2687
124	HD 2985	167	CL1705/HD2687
125	HD 2987	168	CL264//CL1633/CNO-601
126	HD 3043	169	CL264//CL1633/CNO-601
127	HD 2329	170	CL264//CL1633/CNO-601
171	EGPSN-36/PBW 343	174	CL2596/K9451/CL882//HD2009
172	EGPSN-36/PBW 343	175	CL2596/K9451/CL882//HD2009
173	EGPSN-36/PBW343		

Table S2: List of 19 subset genotypes screened under hydroponics and pot

SN	Table S1	Pedigree/Genotypes	Weight (gm)
**On the basis of mean shoot dry weight**			
1	154	HD2824/VL796	1.88
2	144	DL672/P66.270//DE894/3/CUMYN	1.12
3	151	HD2687/HP1896//WH542	1.51
4	152	CSW44	1.195
5	84	HD2967/DBW17	0.195
6	128	HD 3090	0.31
7	5	HD2953/HS365	0.265
**On the basis of mean Nitrogen efficient**			
8	86	HD2967/HD3024	0.775
9	78	CL2596/K9451/CL882//HD2009	0.615
10	101	HD2967/DT2761	0.595
11	114	PBW 621-50	0.08
12	51	PBW343/PICCI LOCAL//RL6080	0.11
13	92	HD2967/WH1073	0.11
**On the basis of mean root dry weight**			
14	173	EGPSN-36/PBW 343	0.1875
15	171	EGPSN-36/PBW 343	0.1435
16	143	DL849/YSCN-08	0.186
17	89	HD2967/DBW56	0.0165
18	71	DL5/PBW343//HD2891	0.019
19	99	HD2967/HD3035	0.0235

Table S3: Clustering pattern of 175 spring wheat genotypes (Table S1) on the basis of morpho-physiological traits for NUE under high nitrogen (HN)

Cluster	Genotype No.
I (160)	83, 100, 39, 47, 20, 85, 73, 59, 1, 30, 6, 21, 165, 26, 18, 17, 56, 91, 34, 13, 41, 36, 60, 95, 67, 98, 94, 58, 104, 62, 22, 3, 50, 49, 70, 4, 65, 64, 52, 158, 99, 45, 66, **5**, 106, 11, 87, 108, 14, 24, 10, 71, 156, 75, 74, 93, 168, 25, 53, 103, 92, 76, 48, 63, 136, 15, 8, 27, 28, 123, 51, 72, 54, 42, 61, 82, 43, 81, 33, 55, 101, 46, 77, 90, 111, 102, 35, 2, 130, 96, 16, 29, 148, 164, 114, 89, 88, **84**, 150, 124, 38, 131, 163, 139, 135, 167, 19, 105, 80, 147, 7, 137, 32, 129, 69, 57, 142, 117, 37, 157, 44, 132, 109, 97, 118, 121, 78, 175, 116, 115, 107, 133, 169, 149, 159, 40, 68, 141, **128**, 122, 112, **152**, **144**, 125, 146, 113, 23, 138, 12, 126, 170, 134, 31, 174, 119, 140, 86, 145, 110, 160
II (1)	127
III (1)	162
IV (11)	143, 153, 166, 155, 173, 172, 9, 171, **151**, 161, 120
V (1)	**154**
VI (1)	79

() indicate numbers of genotypes accommodated in the cluster

Table S4: Clustering pattern of 175 spring wheat genotypes (Table S1) on the basis of morpho-physiological traits for NUE under low nitrogen (LN)

Cluster	Genotype No.
I (136)	74, 106, 65, 118, 39, 11, 169, 141, 29, 167, 164, 128, 109, 175, 163, 138, 165, 18, 69, 143, 131, 75, 146, 156, 105, 129, 149, 113, 45, 134, 104, 132, 71, 8, 10, 44, 145, 117, 33, 25, 84, 107, 130, 35, 37, 171, 111, 68, 12, 127, 172, 159, 115, 27, 13, 5, 120, 90, 126, 40, 160, 123, 152, 137, 155, 7, 153, 124, 34, 157, 147, 170, 60, 108, 26, 89, 173, 135, 20, 116, 17, 62, 53, 2, 16, 9, 77, 148, 32, 59, 52, 67, 73, 6, 158, 64, 70, 142, 61, 22, 21, 43, 31, 144, 122, 139, 30, 125, 46, 47, 63, 24, 42, 133, **51, 92,** 66, 50, 140, 174, 15, 136, 55, 57, 150, 76, 121, 151, 28, 23, 80, 154, 1, 19, 110, **114**
II (22)	41, 161, 79, 94, 98, 85, 103, 88, **101**, **78**, 99, 100, 102, 83, 82, 81, 87, 97, 3, 93, 38, 36
III (10)	56, 58, 54, 166, 72, 168, 48, 162, 96, 14
IV (1)	119
V (1)	112
VI (1)	91
VII (1)	95
VIII (1)	49
IX (1)	**86**
X (1)	4

() indicate numbers of genotypes accommodated in cluster

Table S5: Average intra and inter cluster D^2 values of different clusters under HN

Cluster	I	II	III	IV	V	VI
I	**10.9**					
II	18.21	**0**				
III	19.26	12.45	**0**			
IV	25.94	11.91	15.01	**13.22**		
V	39.44	26.79	24.27	21.86	**0**	
VI	22.87	26.03	21.15	29.89	37.34	**0**

Table S6: Average intra and inter cluster D^2 values of different clusters under LN

Cluster	I	II	III	IV	V	VI	VII	VIII	IX	X
I	**8.41**									
II	17.97	**9.07**								
III	13.45	25.72	**10.4**							
IV	12.8	13.6	22.21	**0**						
V	15.93	13.7	25.54	3.54	**0**					
VI	13.18	15.02	15.77	18.38	20.83	**0**				
VII	21.9	14.32	25.84	23.11	24.19	10.84	**0**			
VIII	18.77	24.58	15.25	27.28	30.16	10.94	18.33	**0**		
IX	26.53	13.73	33.06	23.39	22.97	20.2	14.01	27.89	**0**	
X	14.24	24.64	15.71	18.81	21.65	19.43	27.94	22.38	32.94	**0**

Table S7: Eigen vectors for different NUE traits under HN

	PC1	PC2	PC3	PC4	PC5	PC6	PC7	PC8	PC9	PC10
SDW	**0.452**	0.007	-0.166	0.140	-0.023	-0.096	**0.352**	0.261	**-0.739**	-0.013
RDW	**0.258**	**-0.530**	0.057	0.074	0.334	**-0.612**	**-0.395**	-0.016	0.029	-0.012
MRL	0.156	**-0.470**	0.009	**-0.504**	**-0.706**	0.037	-0.014	-0.005	0.003	-0.003
PH	0.184	0.262	-0.291	**-0.792**	**0.428**	-0.015	-0.036	0.018	0.008	0.001
R:S	-0.076	**-0.595**	0.206	-0.071	**0.453**	**0.518**	**0.344**	0.012	-0.026	0.012
N%	0.131	0.178	**0.640**	-0.115	0.010	0.140	**-0.319**	**0.637**	-0.029	-0.020
gN	**0.463**	0.071	0.044	0.112	0.003	**0.324**	-0.229	**-0.345**	0.035	**-0.699**
NUpE	**0.463**	0.069	0.043	0.112	-0.003	**0.309**	**-0.254**	-0.309	0.013	**0.714**
NUtE	-0.123	-0.182	**-0.635**	0.164	-0.007	**0.348**	**-0.459**	**0.435**	-0.013	-0.016
NUE	**0.452**	0.006	-0.162	0.150	-0.025	-0.069	**0.413**	**0.348**	**0.670**	0.008

Table S8: Eigen vectors for different NUE traits under LN

	PC1	PC2	PC3	PC4	PC5	PC6	PC7	PC8	PC9	PC10
SDW	**0.433**	0.167	0.022	-0.024	-0.124	-0.275	-0.171	-0.028	**0.404**	**-0.707**
RDW	**0.426**	0.152	0.181	-0.092	-0.060	-0.266	-0.136	-0.125	**-0.803**	-0.001
MRL	0.197	-0.032	**-0.491**	**-0.504**	**0.682**	-0.005	-0.011	-0.007	0.000	0.000
PH	0.126	0.229	0.114	**0.743**	**0.605**	0.029	-0.027	-0.002	0.001	0.000
R:S	-0.020	-0.075	**0.844**	**-0.376**	0.333	0.077	0.026	0.017	0.151	0.000
N%	0.080	**-0.643**	0.000	0.128	0.029	0.164	**-0.715**	-0.154	-0.005	0.000
gN	**0.432**	-0.154	-0.002	0.046	-0.095	**0.499**	**0.430**	**-0.585**	0.054	-0.001
NUpE	**0.435**	-0.146	0.005	0.035	-0.091	**0.391**	0.105	**0.782**	-0.062	0.002
NUtE	-0.082	**0.635**	-0.020	-0.155	-0.079	**0.584**	**-0.462**	-0.069	-0.009	0.000
NUE	**0.433**	0.167	0.023	-0.024	-0.124	-0.276	-0.170	-0.032	**0.402**	**0.708**

SDW: Shoot dry weight (gm), RDW: Root dry weight (gm), TDW: Total dry weight (gm), R:S: Root- shoot ratio, MRL: Maximum root length (cm), N%: Nitrogen percentage in shoot (%), gN: Gram N in shoot (gm), NUpE: Nitrogen uptake efficiency, NUtE: Nitrogen utilization efficiency, NUE: Nitrogen use efficiency
